# Supplementary material for: MetaQTL: a package of new computational methods for the meta-analysis of QTL mapping experiments
Source: BMC Bioinformatics. 2007 Feb 8;8:49. doi: 10.1186/1471-2105-8-49 (PMC1808479; doi:10.1186/1471-2105-8-49)
Supplement: Additional File 7 — MetaQTL Package : jar file and tutorial. This Zip archive contains both the MetaQTL JAR file and the files of the tutorial. [file 1471-2105-8-49-S7.zip › org.inra.metaqtl/doc/org/metaqtl/main/CmdLineParser.Option.StringOption.html]

CmdLineParser.Option.StringOption


|  |  |  |
| --- | --- | --- |
| **Overview** \| **Package** \| **Class** \| **Use** \| **Tree** \| **Deprecated** \| **Index** \| **Help** | |  |
| **PREV CLASS**   **NEXT CLASS** | **FRAMES**    **NO FRAMES**     **All Classes** |  |
| SUMMARY: NESTED \| FIELD \| CONSTR \| METHOD | DETAIL: FIELD \| CONSTR \| METHOD |  |


---


## org.metaqtl.main Class CmdLineParser.Option.StringOption

```
java.lang.Object
  org.metaqtl.main.CmdLineParser.Option
      org.metaqtl.main.CmdLineParser.Option.StringOption
```

**Enclosing class:**: CmdLineParser.Option

---

``` public static class CmdLineParser.Option.StringOption extends CmdLineParser.Option ```

An option that expects a string value

---

**Nested Class Summary**

**Nested classes/interfaces inherited from class org.metaqtl.main.CmdLineParser.Option**

`CmdLineParser.Option.BooleanOption, CmdLineParser.Option.DoubleOption, CmdLineParser.Option.IntegerOption, CmdLineParser.Option.LongOption, CmdLineParser.Option.StringOption`


**Constructor Summary**

`CmdLineParser.Option.StringOption(char shortForm,
java.lang.String longForm)`

`CmdLineParser.Option.StringOption(java.lang.String longForm)`


| **Method Summary** | |
| --- | --- |
| `protected  java.lang.Object` | `parseValue(java.lang.String arg, java.util.Locale locale)`             Override to extract and convert an option value passed on the command-line |

**Methods inherited from class org.metaqtl.main.CmdLineParser.Option**

`getValue, longForm, shortForm, wantsValue`

**Methods inherited from class java.lang.Object**

`clone, equals, finalize, getClass, hashCode, notify, notifyAll, toString, wait, wait, wait`

**Constructor Detail**

### CmdLineParser.Option.StringOption

```
public CmdLineParser.Option.StringOption(char shortForm,
                                         java.lang.String longForm)
```

---


### CmdLineParser.Option.StringOption

```
public CmdLineParser.Option.StringOption(java.lang.String longForm)
```


**Method Detail**

### parseValue

```
protected java.lang.Object parseValue(java.lang.String arg,
                                      java.util.Locale locale)
```

:   **Description copied from class: `CmdLineParser.Option`**
:   Override to extract and convert an option value passed on the
    command-line

    :   **Overrides:**: `parseValue` in class `CmdLineParser.Option`


---


|  |  |  |
| --- | --- | --- |
| **Overview** \| **Package** \| **Class** \| **Use** \| **Tree** \| **Deprecated** \| **Index** \| **Help** | |  |
| **PREV CLASS**   **NEXT CLASS** | **FRAMES**    **NO FRAMES**     **All Classes** |  |
| SUMMARY: NESTED \| FIELD \| CONSTR \| METHOD | DETAIL: FIELD \| CONSTR \| METHOD |  |


---
